# Staged Contouring of the Aesthetically Challenged Female Torso

**DOI:** 10.1093/asjof/ojag138.007

**Published:** 2026-07-24

## Abstract

**Goals/Purpose:**

Patients with a “challenged” female torso—characterized by high BMI, significant lipodystrophy, poor tissue quality, prior surgical deformity, or combined breast–torso disharmony—often experience suboptimal outcomes and increased complication risk with single-stage body contouring. Single-stage procedures may expose these patients to flap ischemia, skin necrosis, wound breakdown, and costly medical complications requiring hospitalization and secondary reconstruction. Our staged contouring aims to optimize aesthetic results while minimizing morbidity by dividing torso, breast, and arm reshaping into multiple physiologically tolerable procedures. This study describes this staged approach, outlines its technical principles, and highlights the rationale for staging in high-risk aesthetic torso patients.

**Methods/Technique:**

This surgical approach emphasizes strategic sequencing of procedures and reduced operative stress to safely reshape the torso in patients who would otherwise be poor candidates for single-stage transformation. Stage 1 consists of a modified circumferential torso-abdominoplasty, a wide rectus abdominis fascia advancement (WRAFA) abdominal wall repair, perforator-sparing liposuction, excisional puboplasty. A second stage could include mastopexy with orthotopic auto-augmentation or breast implants, and fat grafting when appropriate. A brachioplasty or a bra-line torsoplasty can be included in this stage. In patients desiring gluteal contouring with a Brazilian buttock lift, the third and final stage consists of fat grafting to the gluteal subcutaneous layer, with heterotopic breast auto-augmentation (fat grafting). Minor residual areas of liposuction can be done at this stage. Staging allows each region to be treated under ideal tension and vascular conditions, producing a more controlled, predictable aesthetic outcome.

**Results/Complications:**

Eight patients successfully underwent our staged approach to complex body contouring. Ages ranged from 28 to 60. BMI cutoff was no greater than 36. Total surgical time was approximately 7 hours for each patient. Figure 1 demonstrates a middle-aged woman who successfully underwent three-stage body contouring with an excellent aesthetic outcome. All surgeries were performed in an office-based surgical center. This staged approach is associated with:

Advantages

- Lower complication rates due to reduced operative time per stage, less undermining, and preservation of perforators.
- Markedly decreased risk of tissue necrosis, seroma, and wound dehiscence—particularly important in patients with compromised soft tissue.
- Reduced systemic stress (blood loss, fluid shifts, infection risk), enabling outpatient management for most stages.
- Superior aesthetic outcomes, given the ability to contour progressively with improved tissue compliance at each stage.
- Avoidance of catastrophic complications (shock, infection, major bleeding) that may require ICU admission, embolization, abscess drainage, or prolonged hospitalization costing upward of several hundred thousand dollars.

Potential Disadvantages

- Increased overall cost and treatment duration.
- Requirement for patient motivation across multiple procedures.
- Longer global treatment timeline.

However, long-term benefits of staging far outweigh these limitations—especially when compared with the morbidity of flap loss, open wounds requiring VAC therapy, skin grafting, flap reconstruction, or prolonged healing by secondary intention.

**Conclusion:**

The aesthetically challenged female torso—often marked by poor tissue quality, significant lipodystrophy, and multidimensional contour deformity—benefits substantially from a staged surgical approach. This technique provides a safe, optimized methodology that reduces major complications while achieving superior long-term contour. By distributing surgical stress, minimizing undermining, and sequencing contouring thoughtfully across three stages, surgeons can deliver transformative, reliable outcomes in a population that historically experiences high complication rates with single-stage operations.

The staged contouring model should be considered a best-practice strategy for complex female torso aesthetics, particularly in high-risk or revision-prone patients.

